# Clinicopathological and molecular comparison of eosinophilic solid and cystic renal cell carcinoma and *TFEB* ‐amplified renal cell carcinoma: a comprehensive study of 15 cases

**DOI:** 10.1111/his.70261

**Published:** 2026-08-13

**Authors:** Hayley Zullow, Melissa Y. Tjota, Jung Woo Kwon, Tatjana Antic

**Affiliations:** ^1^ Department of Pathology The University of Chicago Chicago Illinois USA

**Keywords:** Eosinophilic solid and cystic renal cell carcinoma, renal tumorigenesis, *TFEB*‐amplified renal cell carcinoma

## Abstract

**Aims:**

Eosinophilic solid and cystic renal cell carcinoma (ESC‐RCC) and *TFEB*‐amplified renal cell carcinoma (*TFEB*‐amplified RCC, a subset of *TFEB‐*altered RCC) are rare eosinophilic renal cell tumours that can be difficult to distinguish on the basis of morphology and immunohistochemistry alone. In this study, we compared the clinicopathological and molecular features of these entities to identify distinguishing characteristics.

**Methods and results:**

Nine cases of ESC‐RCC and six cases of *TFEB*‐amplified RCC from our institutional archives were evaluated for clinical, morphological, immunohistochemical and molecular features. Despite morphological overlap, each entity exhibited somewhat distinctive features. ESC‐RCC frequently exhibited solid and cystic architecture with tightly packed tubules, flocculent to coarsely granular cytoplasm with basophilic stippling and prominent admixed macrophages. In comparison, *TFEB*‐amplified RCC more frequently demonstrated solid growth of pseudopapillae and loose tubules, dense eosinophilic granular cytoplasm without basophilic stippling, focally apical nuclei and extracellular magenta globules. However, neither morphology nor immunohistochemistry was sufficiently specific to completely distinguish these entities. Ancillary molecular testing identified pathogenic *TSC1* or *TSC2* gene variants in ESC‐RCC and *TFEB* gene locus amplification in *TFEB*‐amplified RCC.

**Conclusions:**

Although ESC‐RCC and *TFEB*‐RCC share overlapping morphological and immunohistochemical features, each demonstrates somewhat characteristic histological findings. Definitive distinction requires molecular testing to identify the underlying molecular alterations. As molecular testing assumes an increasingly important role in RCC diagnosis and classification, recognition of these rare entities and their overlapping and distinguishing features is essential for appropriate testing and accurate diagnosis.

## Graphical abstract

This study supports the need for ancillary testing to diagnose ESC‐RCC versus *TFEB*‐amplified RCC, as neither morphology nor immunohistochemistry is sufficiently specific to distinguish these entities. The underlying molecular drivers in each tumour are pathogenic *TSC1* or *TSC2* gene variants in ESC‐RCC and *TFEB* gene amplification in *TFEB*‐amplified RCC.

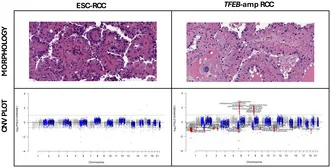

**Abbreviations**
AJCC: American Joint Committee on Cancer
ESC‐RCC: eosinophilic solid and cystic renal cell carcinoma
FISH: fluorescence in situ hybridization
H&E: haematoxylin and eosin
HIPAA: Health Insurance Portability and Accountability Act
IHC: immunohistochemical
NGS: next‐generation sequencing
RCC: renal cell carcinoma
WHO: World Health Organization

## Introduction

Over the past half century, the classification system of renal cell carcinoma (RCC) has changed multiple times to reflect our evolving understanding of renal cell tumour biology.^1^, ^2^ The most recent World Health Organization (WHO) classification defines six groups of renal cell tumours: clear cell, papillary, oncocytic and chromophobe, collecting duct, molecularly defined and other RCC. Clear cell, papillary and chromophobe RCC account for approximately 95% of renal cell tumour diagnoses.^3^, ^4^ These more common RCC subtypes have characteristic morphology and immunohistochemical (IHC) staining patterns, and are most often diagnosed without further ancillary testing.

In contrast, rarer RCC subtypes, especially eosinophilic or oncocytic renal tumours, may exhibit significant morphological and immunophenotypic overlap and frequently require advanced testing modalities for definitive diagnosis.^5^, ^6^ Though ancillary testing is often unavoidable in these cases, identifying specific morphological and IHC features of rare RCC subtypes may guide more effective work‐up and diagnosis of rare renal cell tumours, especially when diagnostic material is limited. Therefore, despite the expanding role of molecular testing in the diagnosis and classification of renal cell tumours, it remains critical to fully characterize and recognize specific features of rare RCC subtypes to improve diagnosis, treatment and outcomes for patients with these tumours.^7^, ^8^

Eosinophilic solid and cystic renal cell carcinoma (ESC‐RCC) and *TFEB*‐amplified renal cell carcinoma (*TFEB*‐amplified RCC, a subset of *TFEB‐*altered RCC) are rare eosinophilic RCC subtypes that are both associated with TSC/mTOR pathway dysregulation and can be difficult to distinguish on the basis of morphology and immunohistochemistry alone.^9^, ^10^, ^11^, ^12^, ^13^ Grossly, both entities present as well‐circumscribed tumours with a yellow cut surface with or without areas of haemorrhage and necrosis; histologically, these tumours are composed of large cells with abundant granular eosinophilic cytoplasm, large nuclei and prominent nucleoli.^14^ Furthermore, though it has been proposed that these entities can be distinguished by differential IHC staining for Melan‐A and CK20, the reported utility of this strategy is variable.^15^, ^16^, ^17^, ^18^ As a result, both ESC‐RCC and *TFEB*‐amplified RCC may remain in the differential diagnosis for an eosinophilic tumour even after morphological and IHC work‐up. The distinction between these entities is critical, as ESC‐RCC is reported to have a better prognosis than *TFEB*‐amplified RCC and may have implications on clinical course and treatment strategy.^19^, ^20^, ^21^

Existing comparative studies of ESC‐RCC and *TFEB*‐amplified RCC remain limited due to the rarity of both entities, and due to the scarcity of papers that compare these entities directly using the same criteria. In this study, we compare the clinical, morphological, IHC and molecular profiles of nine ESC‐RCC cases and six *TFEB*‐amplified RCC cases from a single institution. In addition to expanding published knowledge of these uncommon renal cell tumours, we demonstrate the use of hybrid‐capture next‐generation sequencing (NGS) to identify the characteristic molecular alterations underlying each entity, including detection of *TFEB* gene locus amplification through copy‐number analysis. Using this integrated clinicopathological and molecular approach, we aimed to define features that facilitate recognition of these tumours while emphasizing the role of molecular testing in establishing a definitive diagnosis when morphological and IHC findings are equivocal.

## Methods

### Study Population

This study was approved by the Institutional Review Board at The University of Chicago under protocol IRB‐24‐1223. We retrospectively reviewed The University of Chicago Department of Pathology database from 2005 to 2025 and identified unclassified or unusual eosinophilic renal tumours suspicious for re‐classification as ESC‐RCC or *TFEB*‐amplified RCC, as well as tumours with established diagnoses of ESC‐RCC or *TFEB*‐amplified RCC. Seven cases included in this study (ESC‐RCC cases I, IIA, IIB, III, IV, V, and *TFEB*‐amplified RCC case II) have been reported previously.^13^, ^14^ ESC‐RCC cases were initially given diagnoses of ESC‐RCC (*n* = 1), papillary RCC (*n* = 1) and unclassified RCC (*n* = 7). *TFEB*‐amplified RCC cases were initially diagnosed as sarcomatoid RCC (*n* = 1), renal oncocytoma (*n* = 1) and unclassified RCC (*n* = 4). Clinical data were obtained through electronic medical record review.

### Pathological Analysis and Immunohistochemical Staining

For each case, the original pathological diagnosis, haematoxylin and eosin (H&E)‐stained slides and IHC stains were reviewed by a genitourinary pathologist at The University of Chicago. IHC stains were performed in the clinical laboratory at The University of Chicago using autoimmunostainers (Leica BOND‐III; Leica Biosystems, Buffalo Grove, IL and BenchMark XT Ventana; Roche, Tucson, AZ) with appropriate controls. The following panel of IHC stains was performed: CK7 (COV‐TL 12/30; Dako, Carpinteria, CA), CK20 (KS20.8; Dako, Carpinteria, CA), Vimentin (V9; Dako, Carpinteria, CA), racemase (P504S, CPK 200 AK, BK, CK; Biocare Medical, Concord, CA), CD10 (56C6; Leica NCL, Buffalo Grove, IL), SDHB (21A11AE7; Abcam, Cambridge, MA), Melan‐A (A103; Dako, Carpinteria, CA) and PD‐L1 (22C3; Agilent, Santa Clara, CA).

### Next‐Generation Sequencing

Sufficient material was available for NGS in 5 of 9 ESC‐RCC cases (I, IIA, III, V and VIII) and 6 of 6 *TFEB‐*amplified RCC cases (I–VI). ESC‐RCC case IIB failed multiple attempts to sequence. ESC‐RCC case IV was not sequenced, as it occurred in a patient with tuberous sclerosis and displayed the typical morphological features and IHC phenotype of ESC‐RCC. From the cases that were sequenced, a representative formalin‐fixed, paraffin‐embedded block was selected for NGS on The University of Chicago Medicine OncoPlus (UCM‐OncoPlus) panel, a hybrid‐capture panel targeting 1005 cancer‐associated genes with 168 clinically reported genes. DNA extraction, DNA quantification, library preparation and NGS were performed as described previously.^22^

Data analysis was performed on a HIPAA (Health Insurance Portability and Accountability Act)‐compliant high‐performance computing system (Center for Research Informatics, University of Chicago) using an in‐house developed bioinformatics pipeline. Copy number results were calculated using a combination of CNVkit 14 software^23^ and additional in‐house intra‐run normalization to eliminate run‐specific artefacts, by comparison with a pooled cohort of clinical controls. Focal gene‐level and chromosome‐level changes were called using a combination of thresholds and manual interpretation, as described previously.^22^, ^23^, ^24^ The specific thresholds used in this study were log_2_ (fold change) ≥ 1.5 for amplification and log_2_ (fold change) between 1.0 and 1.5 for equivocal amplification, consistent with thresholds used previously.^25^ Somatic variant calls were inspected using Integrated Genomics Viewer (IGV; Broad Institute, MIT Harvard, Cambridge, MA). Variant review was performed by molecular pathologists and included filters based on population frequencies (the 1000 Genomes Project, https://www.internationalgenome.org/), frequencies in cancer variant databases (COSMIC: Catalogue Of Somatic Mutations In Cancer, https://cancer.sanger.ac.uk/cosmic, and cBioPortal, https://www.cbioportal.org/), and coding effects.

## Results

### Clinical Demographics

Retrospective review and molecular profiling of previously unclassified or unusual eosinophilic renal cell tumours identified nine ESC‐RCC cases (*n* = 9) and six *TFEB*‐amplified RCC cases (*n* = 6) from our institutional archives (Table 1). Two ESC‐RCC cases (cases IIA and IIB) arose in a single patient who underwent right radical nephrectomy for a right‐sided kidney mass (ESC‐RCC case IIA), and 2 months post‐operatively was found to have a mass in the contralateral kidney (ESC‐RCC case IIB). One of the *TFEB*‐amplified RCC cases is from a patient who had presented with metastatic disease to the brain; approximately 15 years earlier, the patient had undergone resection of a primary renal mass that was diagnosed as ‘sarcomatoid RCC’. The metastatic lesion (*TFEB*‐amplified RCC case I) displayed a similar histological appearance to the primary renal tumour and was sequenced for diagnostic purposes. The ESC‐RCC cohort of 9 cases arose in 8 patients (6 female and 2 male) ranging in age from 33 to 68 years at diagnosis (mean: 50 years). The 6 cases of *TFEB‐*amplified RCC arose in 4 female patients and 2 male patients ranging in age from 53 to 76 years at diagnosis (mean: 66 years). One patient in the ESC‐RCC group had a history of tuberous sclerosis (ESC‐RCC case IV). In the *TFEB*‐amplified group, one patient had a history of Gaucher's disease, type 1 (*TFEB*‐amplified RCC case IV) and 1 patient was suspected to have multiple endocrine neoplasia, type 1, though confirmatory testing was not performed (*TFEB*‐amplified RCC case II). No additional patients were known or suspected to have an underlying hereditary syndrome.

**Table 1:** Patient cohort demographics and tumour characteristics

Cases	Age (years)	Sex	Laterality	Focality	Largest size (cm)	Stage	Original grade	Architecture	Hereditary disease	Original diagnosis
ESC‐RCC										
I*	52	F	R	Unifocal	5.5	T1bNx	3	Solid and cystic	Not tested	Unclassified RCC
IIA*	48	F	R	Multifocal	6	T3bN2	High	Solid and papillary	Not tested	Papillary RCC, type 2
IIB*	49	F	L	Multifocal	2.5	T1aN2	High (3–4)	Solid and papillary	Not tested	Unclassified RCC
III*	33	F	L	Unifocal	1.7	T1aNx	Low	Solid and cystic	Not tested	Unclassified RCC
IV*	47	F	R	Unifocal	3	T1aNx	3	Solid and cystic	TSC	Unclassified RCC
V*	65	F	L	Unifocal	11.5	T3aNx	4	Solid and cystic	Not tested	Unclassified oncocytic RCC
VI	37	F	L	Unifocal	2.2	T1aNx	NA	Solid and cystic	Not tested	Eosinophilic solid and cystic RCC
VII	68	M	R	Unifocal	2	T1aNx	2, focal 3	Solid and cystic	Not tested	Unclassified RCC
VIII	51	M	L	Unifocal	4.1	T3aNx	NA	Solid and cystic	Not tested	Unclassified RCC
TFEB‐amplified RCC										
I	53	M	L	Unifocal	11	T3aNx	4	Solid	Not tested	Sarcomatoid RCC
II*	67	F	R	Unifocal	2.9	T3aNx	NA	Solid and papillary	MEN1 (suspected)	Renal oncocytoma
III	64	F	L	Unifocal	7.6	T3aNx	3, focal 4	Solid and papillary	Not tested	Unclassified RCC
IV	65	F	L	Unifocal	9.1	T3aNx	3	Solid	Gaucher	Unclassified RCC
V	71	M	R	Unifocal	3.7	T1aNx	3	Tubulopapillary	Not tested	Unclassified RCC
VI	76	F	L	Unifocal	7.5	T3aNx	High	Solid and tubulopapillary	Not tested	Unclassified RCC

* Previously published cases.

### AJCC Tumour Staging

Upon resection, *TFEB*‐amplified tumours were larger and more frequently higher in stage than ESC‐RCC tumours (Table 1). ESC‐RCC tumours ranged in size from 1.7 to 11.5 cm in greatest dimension (mean: 4.3 cm). Per the American Joint Committee on Cancer (AJCC), six ESC‐RCC resections revealed pathological stage T1 disease (*n* = 6) and the remaining three resections revealed pathological stage T3 disease (*n* = 3). Lymph nodes were recovered in two ESC‐RCC resections corresponding to a single patient (ESC‐RCC cases IIA and IIB); metastatic carcinoma was identified in >1 regional lymph node in each of the resection specimens. Resected *TFEB*‐amplified tumours ranged in size from 2.9 cm to 11.0 cm in greatest dimension (mean: 7.0 cm). At primary resection, 5 patients with *TFEB*‐amplified RCC had pathological stage T3 disease (*n* = 5) and 1 patient had pathological stage T1 disease (*n* = 1). No lymph nodes were recovered in the resection specimens for *TFEB*‐amplified RCC.

### Morphological Features

Review of H&E slides demonstrated that both entities exhibited shared as well as somewhat distinctive morphological features. Both ESC‐RCC and *TFEB*‐amplified tumours were at least partially solid and composed of polygonal cells with abundant eosinophilic cytoplasm and focally large, high‐grade nuclei with prominent nucleoli (Table 2).

**Table 2:** Histomorphological comparison of ESC‐RCC and *TFEB*‐amplified RCC

Features	ESC‐RCC	TFEB‐amplified RCC
Architecture	Solid and cystic predominantly tubular, with tightly packed small tubules	Solid pseudopapillary to tubular, with large tubules
Cytoplasm	Abundant, eosinophilic, occasionally vacuolated magenta‐blue granules	Abundant, eosinophilic, densely granular
Nucleus	Large, pleomorphic, high‐grade nuclei with prominent nucleoli	Large, pleomorphic, high‐grade nuclei with prominent nucleoli focally apical
Other	Hobnail cells lining cystic areas foamy macrophages	Extracellular magenta globules calcifications

ESC‐RCC was further characterized by solid and cystic architecture, with solid areas populated by small, tightly packed tubules (Figure 1). The abundant eosinophilic cytoplasm of malignant cells in ESC‐RCC had coarse basophilic stippling and was occasionally vacuolated; alternatively, the cytoplasm was flocculent‐appearing (Figure 2). Additional features of ESC‐RCC included hob‐nailing of cells lining cystic spaces and conspicuous foamy macrophages admixed with or clustered within the tumour.

**Figure 1:**
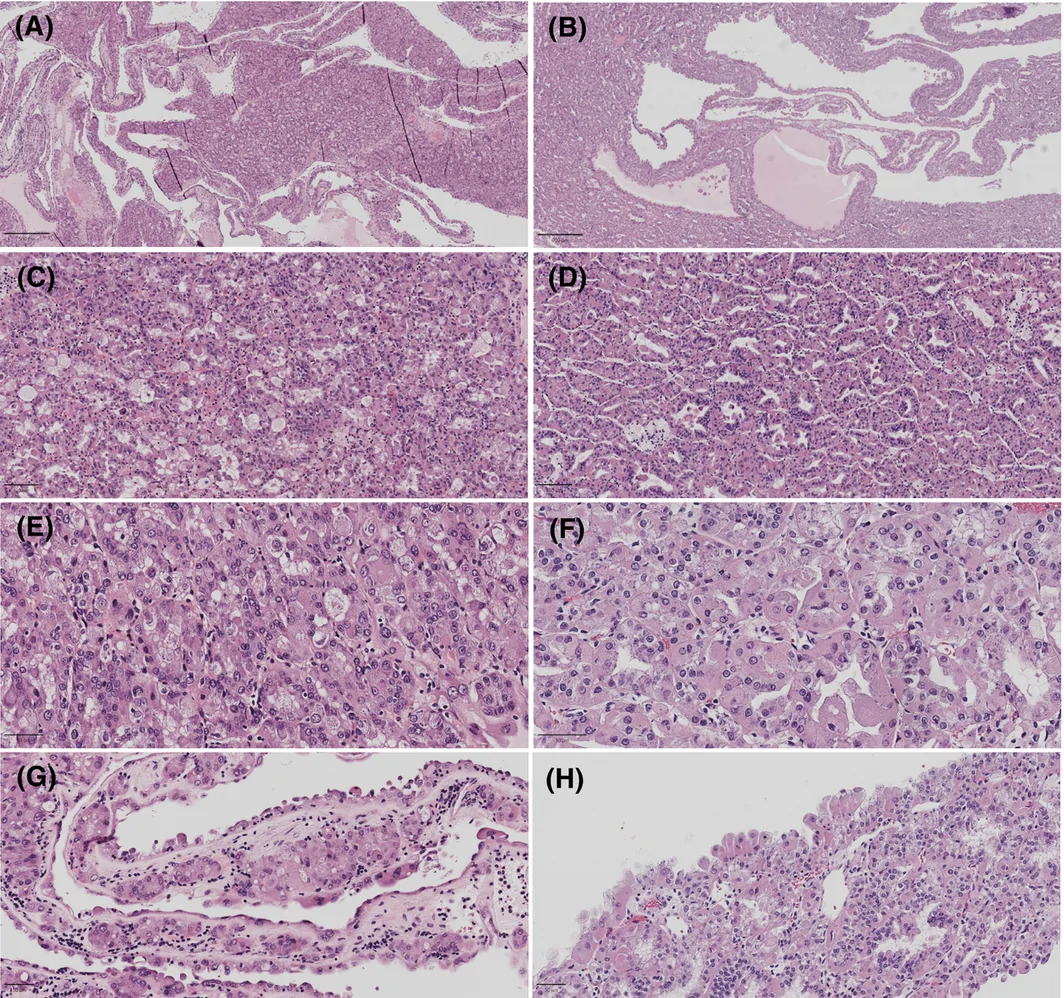
Characteristic morphology of ESC‐RCC in case I (**A, C, E, G**) and case III (**B, D, F, H**). Tumours are solid and cystic (**A, B**), with solid areas comprised of tightly packed, small tubules with admixed foamy macrophages (**C, D**). Malignant cells have abundant eosinophilic cytoplasm with coarse magenta‐blue granules (**E, F**). Cystic spaces are focally lined by hobnailed malignant cells (**G, H**).

**Figure 2:**
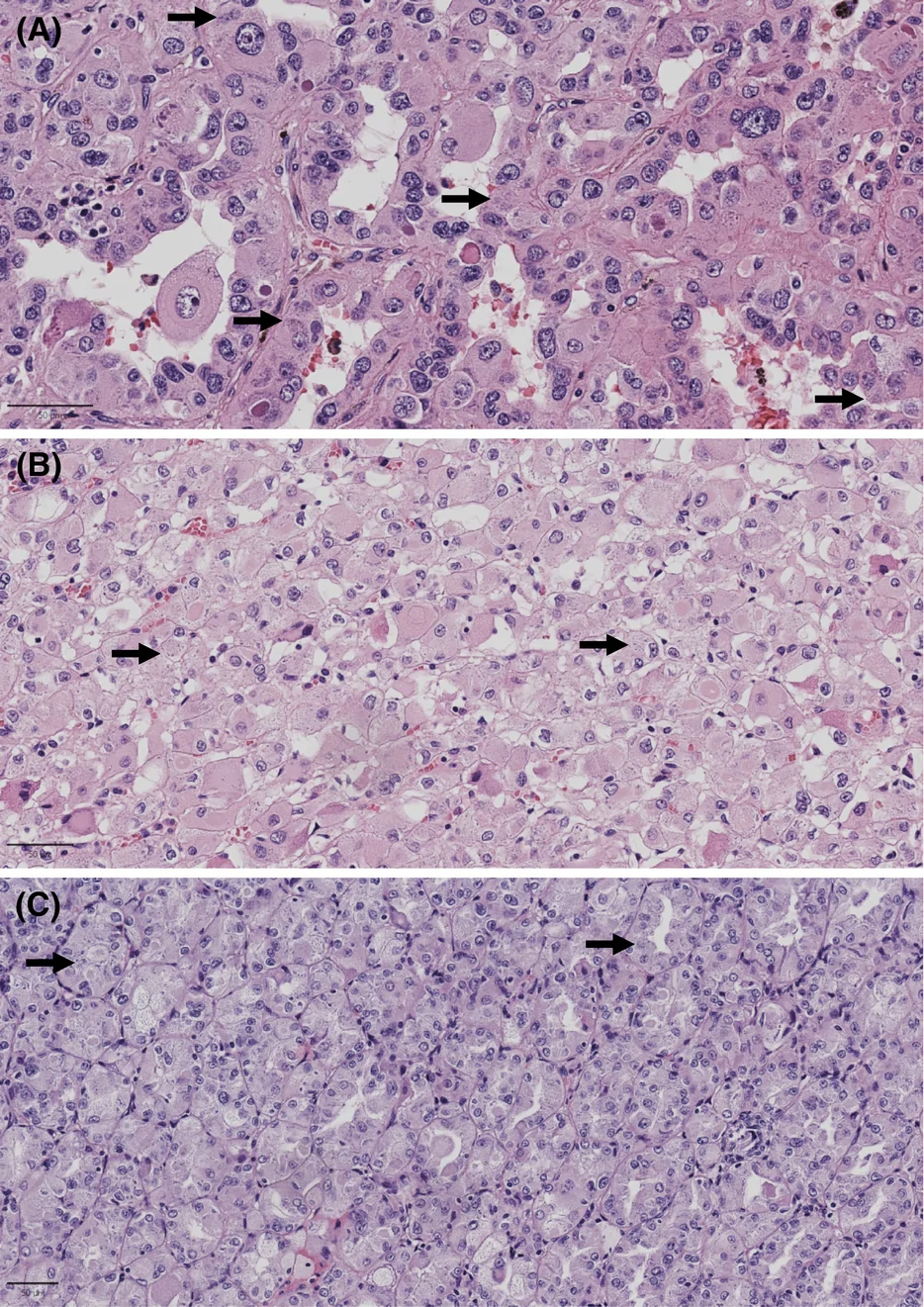
Characteristic nuclear and cytoplasmic features in ESC‐RCC in case II (**A**), case V (**B**) and case VI (**C**). Malignant cells have high‐grade, large and focally pleomorphic nuclei, prominent nucleoli and abundant eosinophilic cytoplasm with basophilic stippling (black arrows).

*TFEB*‐amplified RCC was predominantly solid with areas of alternating tubular and pseudopapillary growth (Figure 3). The tubules were generally larger than those observed in ESC‐RCC and neither macrocystic nor microcystic architecture was present; *TFEB*‐amplified tumour cells predominantly demonstrated dense granular eosinophilic cytoplasm. Basophilic stippling was not identified. Additional findings included apically placed nuclei within pseudopapillary areas, extracellular magenta globules and rare calcifications (Figure S1).

**Figure 3:**
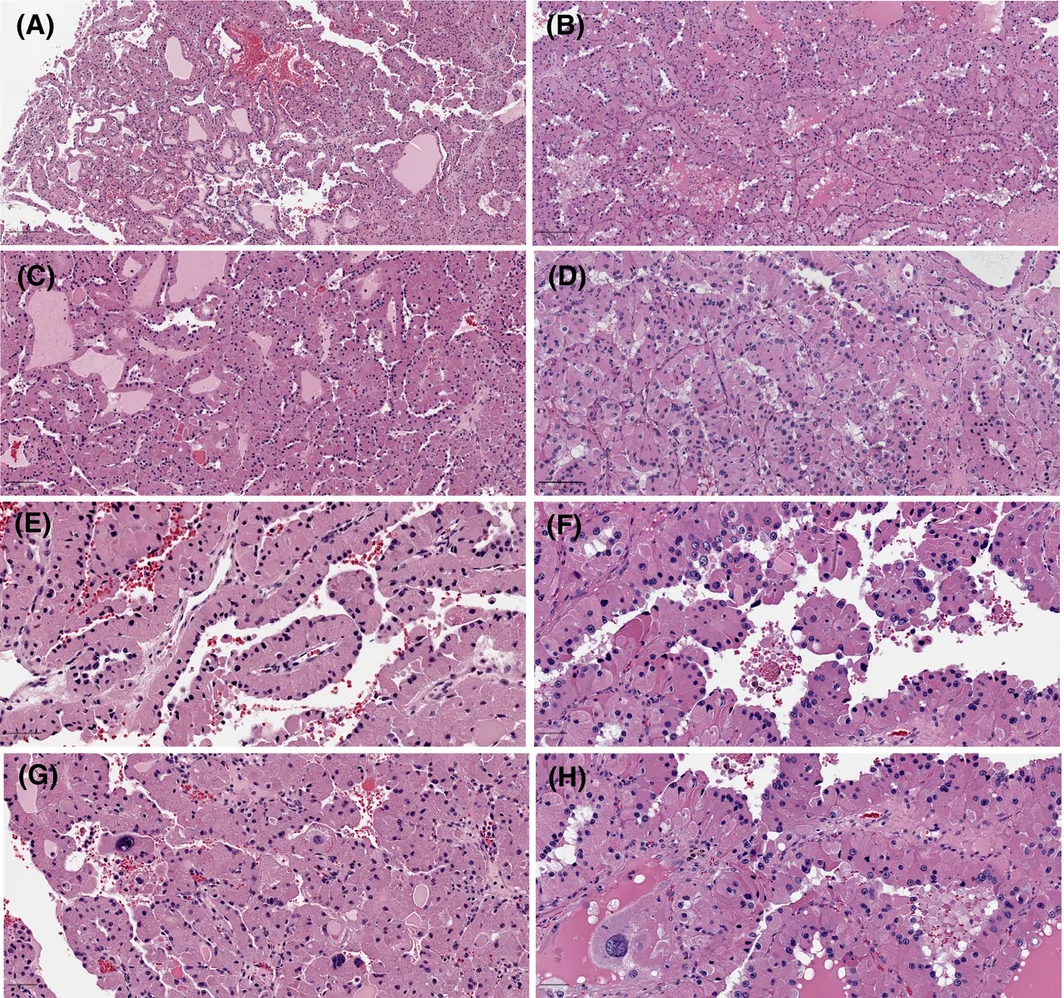
Characteristic morphology of *TFEB*‐amplified RCC in case II (**A, C, E, G**) and case V (**B, D, F, H**). Tumours exhibit variable solid to pseudo‐tubulopapillary architecture and are composed of malignant cells with abundant granular eosinophilic cytoplasm (**A–D**). Nuclei are occasionally apical, predominantly in pseudopapillary areas (**E, F**). Nuclei are large, pleomorphic and focally high‐grade, and extracellular magenta globules are present (**G, H**). [Colour figure can be viewed at wileyonlinelibrary.com]

Overlapping morphological features were observed in selected tumours. ESC‐RCC case VIII exhibited focal pseudopapillary architecture with apically positioned nuclei resembling features more associated with *TFEB*‐amplified RCC (Figure 4A). Conversely, *TFEB*‐amplified RCC case III contained areas with small back‐to‐back tubules and clusters of macrophages reminiscent of ESC‐RCC (Figure 4B).

**Figure 4:**
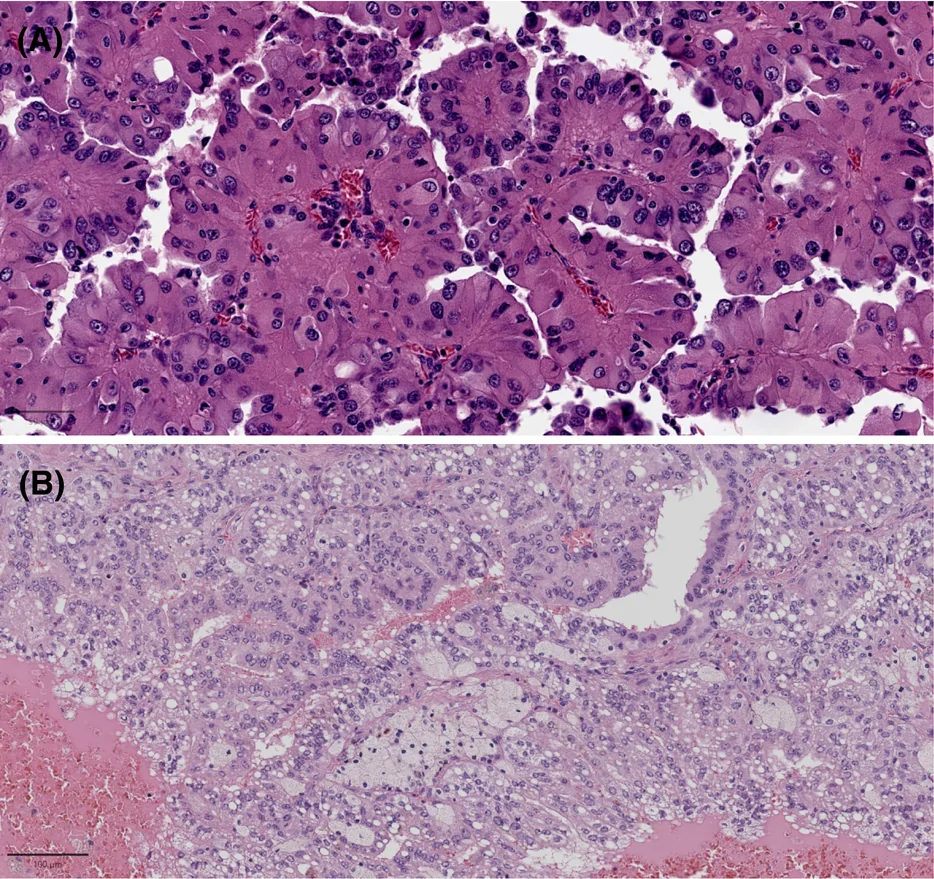
Characteristic features of ESC‐RCC found in *TFEB*‐amplified RCC, and vice versa. ESC‐RCC case VIII exhibits pseudo‐papillary areas with apical nuclei (**A**). *TFEB*‐amplified RCC case III is focally comprised of small tubules of malignant cells with vacuolated cytoplasm and a prominent cluster of macrophages within the tumour (**B**). [Colour figure can be viewed at wileyonlinelibrary.com]

### Immunohistochemistry

Immunohistochemical (IHC) staining was performed using antibodies against vimentin, CK7, CK20, P504S, CD10, SDHB, Melan‐A and PD‐L1 (Table 3). Staining was scored according to percentage of tumour cells demonstrating the appropriate positive staining pattern as follows: 0% (score 0), 1%–5% (score 1), 5%–50% (score 2) and >50% (score 3). Tumours with >5% positive cells were considered positive (corresponding to IHC score ≥2). Both ESC‐RCC and *TFEB*‐amplified RCC exhibited strong, diffuse staining for vimentin and P504S with retained SDHB. CK7 expression was absent or only focally present in both entities. CD10 staining was focal when present. PD‐L1 expression was variable in both cohorts. CK20 staining (>5% positive tumour cells) was identified in 7 of 9 ESC‐RCC cases (78%) and in 3 of 6 *TFEB*‐amplified RCC cases (50%) (*p* = 0.3287). Melan‐A staining (>5% positive tumour cells) was identified in 2 of 9 ESC‐RCC cases (22%) and in 4 of 6 *TFEB*‐amplified RCC cases (66%) (*p* = 0.1357) (Figures S2 and S3).

**Table 3:** Immunohistochemical staining in ESC‐RCC and *TFEB*‐amplified RCC

Case	CK20	MelanA	Vimentin	CK7	P504S	CD10	SDHB	PD‐L1
ESC‐RCC								
I	3	2	3	0	2	0	ND	1
IIA	1	0	3	0	3	0	ND	2
IIB	1	0	3	0	2	0	ND	ND
III	3	0	3	0	2	0	3	3
IV	2	0	2	0	3	1	ND	2
V	3	2	3	0	2	0	3	2
VI	3	0	ND	0	ND	ND	ND	1
VII	3	0	ND	0	ND	ND	ND	0
VIII	2	0	3	ND	3	ND	ND	2
*TFEB*‐amplified RCC								
I	3	3	3	0	3	ND	ND	ND
II	3	2	3	1	ND	ND	ND	1
III	1	1	3	1	3	ND	3	0
IV	0	3	ND	ND	ND	ND	ND	3
V	1	3	ND	ND	ND	ND	ND	3
VI	3	1	3	0	ND	2	3	3

Scoring. 0%–0% staining. 1%–1% to 5%. 2%–5% to 50%. 3%– >50%.

ND, not done.

### Next‐Generation Sequencing

Next‐generation sequencing (NGS) was successfully performed on 5 of 9 ESC‐RCC cases and all 6 *TFEB‐*amplified RCC cases included in this study (Table 4). All sequenced ESC‐RCC cases harboured pathogenic *TSC1* or *TSC2* variants. All *TFEB*‐amplified RCC cases demonstrated focal copy number gain involving chromosome 6p and encompassing the *TFEB* gene locus (Figure 5). Copy number alterations were not identified in any of the ESC‐RCC cases.

**Table 4:** Molecular characterization of ESC‐RCC and *TFEB*‐amplified RCC

Cases	6p amplification with TFEB locus	Pathogenic TSC variant	Variant coding effect	cNomen	pNomen
ESC‐RCC					
I	No	TSC2	Frameshift	NM_000548.4:c.276_279dup	p.P94Afs*33
		TSC2	Frameshift	NM_000548.4:c.938_939del	p.R313Kfs*24
IIA	No	TSC1	Frameshift	NM_000368.4:c.2599_2600insCG	p.Q867Pfs*12
III	No	TSC2	In‐frame deletion	NM.000548.4:c.5238_5255del	p.H1746_R1751del
		TSC2	Splicing	NM_000548.4:c.975+1G>T	p.?
V	No	TSC2	Nonsense	NM_000548.4:c.1635_1636delinsTT	p.E546*
		TSC2	Splicing	NM_000548.4:c.2356‐1G>C	p.?
VIII	No	TSC1	Frameshift	NM_000368.5:c.976_977del	p.L326Tfs*14
*TFEB*‐amplified RCC					
I (m)	Yes	None			
II	Yes	None			
III	Yes	None			
IV	Yes	None			
V	Yes‐equivocal	None			
VI	Yes	None			

**Figure 5:**
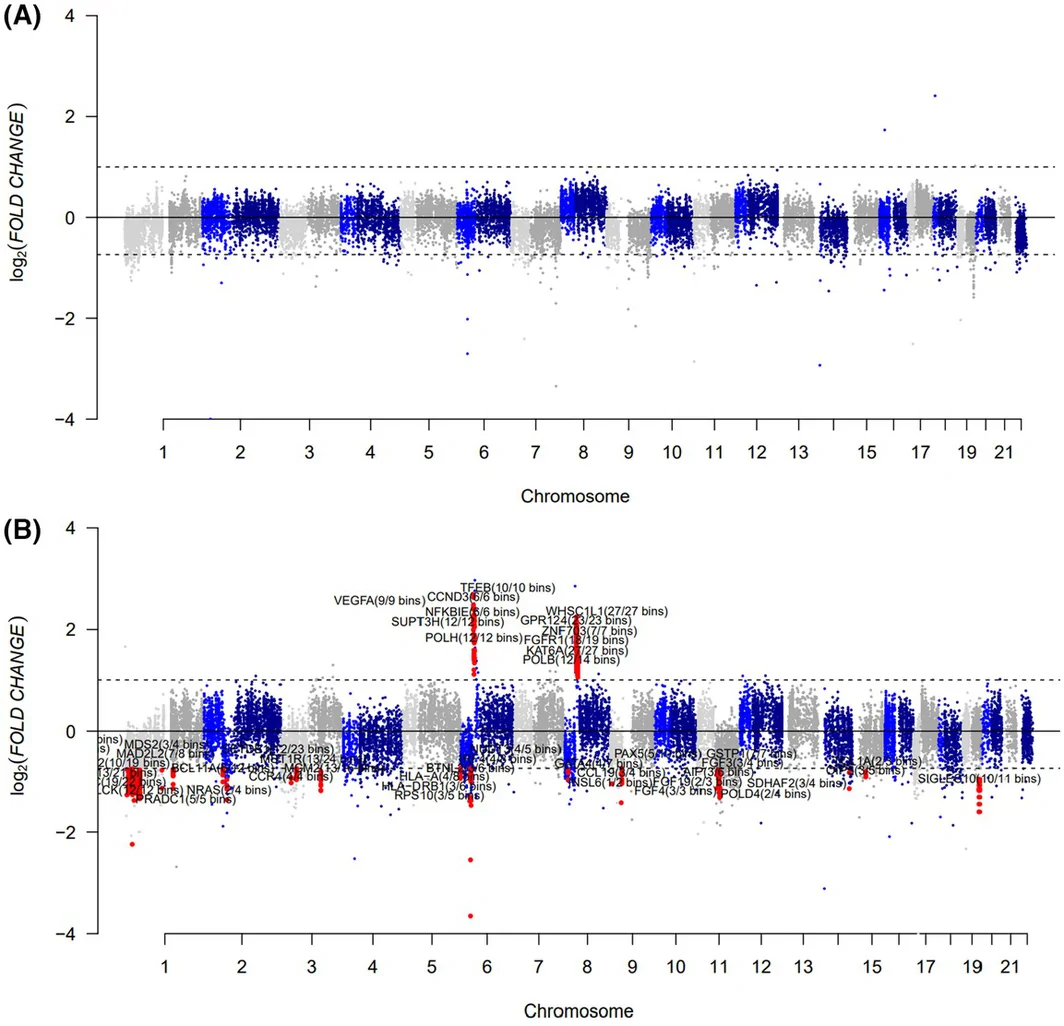
Representative CNV plots. ESC‐RCC case VIII exhibits no significant copy number changes (**A**). *TFEB*‐amplified RCC case IV demonstrates *TFEB* locus amplification on chromosome 6p (**B**). [Colour figure can be viewed at wileyonlinelibrary.com]

### Follow‐Up

Four of the patients in this study were lost to follow‐up (*n* = 4; 2 patients from the ESC‐RCC group corresponding to cases IIA, IIB and III, and 2 patients from the *TFEB‐*amplified RCC group corresponding to cases I and II) (Table 5). One of the ESC‐RCC patients lost to follow‐up had documented concern for metastatic disease to the liver; of note, this patient (corresponding to ESC‐RCC cases IIA and IIB) had known lymph node metastases at the time of tumour resection. One patient with *TFEB*‐amplified RCC lost to follow‐up had documented brain and bony metastases (*TFEB*‐amplified RCC case I) and the other had known recurrent disease in the ipsilateral kidney (*TFEB*‐amplified RCC case II).

**Table 5:** Clinical follow‐up of patient cohorts

Cases	Follow‐up (mo)	Current status	Additional information
ESC‐RCC			
I	231	Alive	NED
IIA	18	Lost to follow‐up	Concern for metastasis (not confirmed)
IIB	14		
III	64	Lost to follow‐up	NED
IV	157	Deceased	Cause of death unknown
V	3	Deceased	Secondary to primary myelofibrosis
VI	49	Alive	NED
VII	18	Deceased	Secondary to cholangiocarcinoma
VIII	38	Alive	NED
*TFEB*‐amplified RCC			
I	205	Lost to follow‐up	Metastatic disease to brain and bone
II	87	Lost to follow‐up	Recurrent disease in right kidney
III	54	Alive	Metastatic disease to lung
IV	36	Alive	Concern for metastasis (not confirmed)
V	17	Alive	NED
VI	1	Alive	Scheduled for nephrectomy

NED, no evidence of disease.

Of the remaining patients not lost to follow‐up, three ESC‐RCC patients were deceased at the time of analysis (*n* = 3; ESC‐RCC cases IV, V, VII). Two of these patients died due to causes unrelated to RCC (ESC‐RCC cases V and VII) and the other patient's cause of death was not documented in our records (ESC‐RCC case IV). The remaining three ESC‐RCC patients that were alive at the time of analysis had follow‐up periods ranging from 38 to 231 months (*n* = 3). These patients did not present with concern for recurrence or metastasis during their respective follow‐up periods. The four *TFEB‐*amplified RCC patients not lost to follow‐up had follow‐up periods ranging from 1 to 54 months (*n* = 4). One of these patients had confirmed metastases (*TFEB*‐amplified RCC case III) and 1 patient was under evaluation due to documented concern for metastatic disease to the lung (*TFEB*‐amplified RCC case IV). Neither of the remaining two *TFEB‐*amplified RCC patients not lost to follow‐up had evidence of disease at the time of this report.

## Discussion

In this study, we compared the clinicopathological and molecular characteristics of nine ESC‐RCC cases and six *TFEB*‐amplified RCC cases from our institutional archives. Although this study was limited by the rarity of these entities and the resulting small cohort size, the clinical characteristics of our cases are consistent with prior reports and further expand the published experience with both entities. Patients with ESC‐RCC were generally younger (mean age, 50 years), demonstrated a female predominance and most presented with stage pT1 disease, consistent with the expected indolent clinical behaviour reported for this entity.^25^, ^26^ In contrast, patients with *TFEB*‐amplified RCC were older at diagnosis (mean age, 66 years), more frequently presented with more advanced stage tumours and were more likely to develop recurrent or metastatic disease during the follow‐up period, supporting previous observations that *TFEB‐*amplified RCC is an aggressive neoplasm.^21^, ^27^, ^28^, ^29^

Although ESC‐RCC and *TFEB*‐amplified RCC demonstrated some morphological overlap, each entity demonstrated somewhat characteristic histological patterns. ESC‐RCC typically exhibited a solid and cystic pattern composed of tightly packed tubules, abundant eosinophilic cytoplasm with coarse basophilic stippling or flocculent appearance, hobnail cells lining cystic spaces and admixed foamy macrophages. In contrast, *TFEB*‐amplified RCC generally demonstrated predominantly solid growth with tubular and pseudopapillary architecture, dense granular eosinophilic cytoplasm lacking basophilic stippling, apically positioned nuclei within pseudopapillary areas, extracellular magenta globules and occasional calcifications.

Importantly, no single morphological feature was entirely specific for either entity. Selected tumours demonstrated overlapping features, including focal pseudopapillary architecture in one ESC‐RCC and focal tightly packed tubules with clustered macrophages in one *TFEB*‐amplified RCC. Furthermore, while basophilic stippling was present in all ESC‐RCC cases and absent in all *TFEB*‐amplified RCC cases, this finding has been reported in other eosinophilic renal neoplasms and therefore should be interpreted in the context of the overall and ancillary findings rather than as an isolated diagnostic feature.^30^, ^31^, ^32^, ^33^ Likewise, psammomatous calcifications, previously reported in up to 50% of ESC‐RCCs, were not identified in our cohort.^27^, ^29^

The IHC staining profiles of these entities also demonstrated considerable overlap. Both entities showed diffuse positive expression of vimentin and P504S with retained SDHB expression and were largely negative or only focally positive for CK7. Although ESC‐RCC more frequently exhibited diffuse CK20 expression with absent or patchy Melan‐A staining, *TFEB*‐amplified RCC more often exhibited the opposite pattern; these differences were not statistically significant. Therefore, CK20 and Melan‐A may provide supportive evidence but are insufficient to reliably distinguish these tumours in isolation.

PD‐L1 expression was variable in both entities, both among tumours and within individual cases. PD‐L1 expression is of particular interest in *TFEB‐*amplified RCC because of its aggressive clinical behaviour and the potential role of immune checkpoint inhibition. Experimental studies have shown that the TFEB protein promotes PD‐L1 expression, facilitating immune evasion and mTOR inhibition resistance.^34^ Consistent with previous reports demonstrating PD‐L1 expression in *TFEB*‐amplified tumours,^35^, ^36^ several tumours in our cohort exhibited strong PD‐L1 staining unrelated to the age of the specimen, despite literature reporting a negative correlation between specimen age and PD‐L1 immunoreactivity^37^, ^38^ Furthermore, a published case report describing a favourable clinical response of *TFEB*‐amplified RCC to anti‐PD‐L1 therapy suggests that immune checkpoint inhibition may represent a therapeutic strategy for selected patients, though additional clinical studies are needed.^39^

Molecular profiling confirmed the defining genomic alterations of both entities. All sequenced ESC‐RCCs harboured pathogenic *TSC1* or *TSC2* variants and every case of *TFEB‐*amplified RCC demonstrated focal amplification of the short arm of chromosome 6 involving the *TFEB* locus.^29^ The protein products encoded by *TSC1/2* and *TFEB* participate in regulation of the PI3K‐AKT–mTOR pathway, which is recurrently altered and up‐regulated in renal tumorigenesis.^2^, ^40^ Pathogenic *TSC1/2* variants resulting in loss of function lead to up‐regulation of mTOR signalling, while *TFEB* amplification results in increased TFEB function downstream of mTOR.^41^ These convergent molecular mechanisms may partially explain the morphological overlap between these entities.^42^

Traditionally, *TFEB* amplification has been established using fluorescence in situ hybridization (FISH). Recent studies have proposed FISH thresholds of >5 copies of *TFEB* to define amplification, with 5–10 copies representing low‐level amplification and >10 copies representing high‐level amplification.^36^ In the present study, *TFEB* amplification was identified using copy‐number analysis generated from routine hybrid capture NGS. NGS panels are routinely used to evaluate copy number gains,^43^, ^44^, ^45^ and have been previously used to diagnose *TFEB*‐amplified RCC. This study demonstrates the feasibility of using NGS to simultaneously identify sequence variants characteristic of ESC‐RCC and focal amplification characteristic of *TFEB*‐amplified RCC.

In summary, morphology and immunohistochemistry remain essential for generating an appropriate differential diagnosis in eosinophilic renal cell tumours. However, due to substantial overlap between ESC‐RCC and *TFEB*‐amplified RCC, molecular characterization is frequently required to establish a definitive diagnosis. Accurate distinction between these entities has important prognostic and potentially therapeutic implications, as ESC‐RCC generally follows an indolent clinical course whereas TFEB‐amplified RCC is associated with more aggressive behaviour. As molecular testing becomes increasingly routine in renal cell tumour diagnosis, integrated clinicopathological and molecular evaluation will be essential for the accurate classification of these uncommon neoplasms.

## Author contributions

Tatjana Antic and Hayley Zullow contributed to the study conception and design. Material preparation, data collection and analysis were performed by all authors (Hayley Zullow, Melissa Y. Tjota, Jung Woo Kwon and Tatjana Antic). The first draft of the manuscript was written by Hayley Zullow and all (Hayley Zullow, Melissa Y. Tjota, Jung Woo Kwon and Tatjana Antic) commented on previous versions of the manuscript. All authors (Hayley Zullow, Melissa Y. Tjota, Jung Woo Kwon and Tatjana Antic) read and approved the final manuscript.

## Conflict of interest statement

All authors have no interests to declare.

## Consent

Waived by the University of Chicago Institutional Review Board (IRB‐24‐1223).

## Supporting information


**Figure S1.** Characteristic features of *TFEB*‐amplified RCC with extracellular magenta globules in case II (A), pseudopapillary areas with apical nuclei in case III (B) and case V (C) and focal calcifications in case V (C).

**Figure S2.** Immunohistochemistry for CK20 in representative cases with strong positive staining (ESC‐RCC case I and *TFEB*‐amplified RCC case II).

**Figure S3.** Fisher's exact test for CK20 and melan‐A immunohistochemistry.

## Data Availability

Data sharing not applicable to this article as no datasets were generated or analysed during the current study.

## References

[1] Caliò A , Marletta S , Brunelli M , Martignoni G . WHO 2022 classification of kidney tumors: What is relevant? An update and future novelties for the pathologist. Pathologica 2022; 115; 23–31. 10.32074/1591-951X-814 PMC10342215 36645397

[2] Linehan WM , Ricketts CJ . The cancer genome atlas of renal cell carcinoma: Findings and clinical implications. Nat. Rev. Urol. 2019; 16; 539–552. 10.1038/s41585-019-0211-5 31278395

[3] Rose TL , Kim WY . Renal Cell Carcinoma: A Review. JAMA 2024; 332; 1001–1010. 10.1001/jama.2024.12848 PMC11790279 39196544

[4] Moch H , Amin MB , Berney DM *et al*. The 2022 World Health Organization classification of Tumours of the urinary system and male genital organs‐part a: Renal, penile, and testicular Tumours. Eur. Urol. 2022; 82; 458–468. 10.1016/j.eururo.2022.06.016 35853783

[5] Udager AM , Mehra R . Morphologic, molecular, and taxonomic evolution of renal cell carcinoma: A conceptual perspective with emphasis on updates to the 2016 World Health Organization classification. Arch. Pathol. Lab Med. 2016; 140; 1026–1037. 10.5858/arpa.2016-0218-RA 27684973

[6] Trpkov K , Hes O , Williamson SR *et al*. New developments in existing WHO entities and evolving molecular concepts: The genitourinary pathology society (GUPS) update on renal neoplasia. Mod. Pathol. 2021; 34; 1392–1424. 10.1038/s41379-021-00779-w 33664427

[7] Matar S , el Ahmar N , Laimon YN *et al*. The role of the pathologist in renal cell carcinoma management. Hematol. Oncol. Clin. North Am. 2023; 37; 849–862. 10.1016/j.hoc.2023.04.014 PMC12543296 37258353

[8] Millan B , Loebach L , Blachman‐Braun R *et al*. Molecular genetics of renal cell carcinoma: A narrative review focused on clinical relevance. Curr. Oncol. 2025; 32; 359. 10.3390/curroncol32060359 PMC12192110 40558302

[9] WHO Classification of Tumours Editorial Board . Eosinophilic solid and cystic renal cell carcinoma. In WHO classification of Tumours: Urinary and male genital Tumours. 5th ed. Lyon, France: International Agency for Research on Cancer, 2022.

[10] WHO Classification of Tumours Editorial Board . TFEB‐altered renal cell carcinoma. In WHO classification of Tumours: Urinary and male genital Tumours. 5th ed. Lyon, France: International Agency for Research on Cancer, 2022.

[11] Kapur P , Brugarolas J , Trpkov K . Recent advances in renal tumors with TSC/mTOR pathway abnormalities in patients with tuberous sclerosis complex and in the sporadic setting. Cancer 2023; 15; 4043. 10.3390/cancers15164043 PMC10452688 37627070

[12] Alesi N , Khabibullin D , Rosenthal DM *et al*. TFEB drives mTORC1 hyperactivation and kidney disease in tuberous sclerosis complex. Nat. Commun. 2024; 15; 406. 10.1038/s41467-023-44229-4 PMC10776564 38195686

[13] Parilla M , Kadri S , Patil SA *et al*. Are sporadic eosinophilic solid and cystic renal cell carcinomas characterized by somatic tuberous sclerosis gene mutations? Am. J. Surg. Pathol. 2018; 42; 911–917. 10.1097/PAS.0000000000001067 29668487

[14] Tjota M , Chen H , Parilla M , Wanjari P , Segal J , Antic T . Eosinophilic renal cell tumors with a TSC and MTOR gene mutations are morphologically and immunohistochemically heterogenous: Clinicopathologic and molecular study. Am. J. Surg. Pathol. 2020; 44; 943–954. 10.1097/PAS.0000000000001457 32091432

[15] Lobo J , Rechsteiner M , Helmchen BM , Rupp NJ , Weber A , Moch H . Eosinophilic solid and cystic renal cell carcinoma and renal cell carcinomas with TFEB alterations: A comparative study. Histopathology 2022; 81; 32–43. 10.1111/his.14663 35403742

[16] Argani P . MiT family translocation carcinomas of the kidney and related entities. Histopathology 2026; 88; 193–213. 10.1111/his.15560 41384707

[17] Trpkov K , Siadat F , Saleeb R . Recent developments in eosinophilic renal neoplasms: what's new, true and important? Histopathology 2026; 88; 166–192. 10.1111/his.70001 PMC12700065 41384711

[18] Alruwaii ZI , Williamson SR , Cheng L , Al‐Obaidy KI . Renal cell tumours with papillary architecture: Evolving concepts, classification and new emerging entities. Histopathology 2026; 88; 149–165. 10.1111/his.70008 41384691

[19] Wang Q , Xu Z , Wang J *et al*. Morphology spectrum and molecular landscape in eosinophilic solid and cystic renal cell carcinoma. Hum. Pathol. 2026; 174; 106141. 10.1016/j.humpath.2026.106141 42055232

[20] Palsgrove DN , Li Y , Pratilas CA *et al*. Eosinophilic solid and cystic (ESC) renal cell Carcinomas Harbor TSC mutations: Molecular analysis supports an expanding clinicopathologic Spectrum. Am. J. Surg. Pathol. 2018; 42; 1166–1181. 10.1097/PAS.0000000000001111 PMC6089659 29975249

[21] Argani P , Reuter VE , Zhang L *et al*. TFEB‐amplified renal cell carcinomas: An aggressive molecular subset demonstrating variable melanocytic marker expression and morphologic heterogeneity. Am. J. Surg. Pathol. 2016; 40; 1484–1495. 10.1097/PAS.0000000000000720 PMC5069163 27565001

[22] Kadri S , Long BC , Mujacic I *et al*. Clinical validation of a next‐generation sequencing genomic oncology panel via cross‐platform benchmarking against established amplicon sequencing assays. J. Mol. Diagn. 2017; 19; 43–56. 10.1016/j.jmoldx.2016.07.012 27836695

[23] Talevich E , Shain AH , Botton T , Bastian BC . CNVkit: Genome‐wide copy number detection and visualization from targeted DNA sequencing. PLoS Comput. Biol. 2016; 12; e1004873. 10.1371/journal.pcbi.1004873 PMC4839673 27100738

[24] Gao J , Aksoy BA , Dogrusoz U *et al*. Integrative analysis of complex cancer genomics and clinical profiles using the cBioPortal. Sci. Signal. 2013; 6; pl1. 10.1126/scisignal.2004088 PMC4160307 23550210

[25] Gupta S , Argani P , Jungbluth AA *et al*. TFEB expression profiling in renal cell carcinomas: Clinicopathologic correlations. Am. J. Surg. Pathol. 2019; 43; 1445–1461. 10.1097/PAS.0000000000001307 PMC6788764 31600176

[26] Trpkov K , Abou‐Ouf H , Hes O *et al*. Eosinophilic solid and cystic renal cell carcinoma (ESC RCC): Further morphologic and molecular characterization of ESC RCC as a distinct entity. Am. J. Surg. Pathol. 2017; 41; 1299–1308. 10.1097/PAS.0000000000000838 28786877

[27] Loghin A , Popelea MC , Todea‐Moga CD *et al*. Eosinophilic solid and cystic renal cell carcinoma‐a systematic review and meta‐analysis. Int. J. Mol. Sci. 2024; 25; 5982. 10.3390/ijms25115982 PMC11172930 38892169

[28] Wu J , Cao CZ , Cui HL *et al*. Prognosis and clinicopathological characters of adult TFEB‐altered renal cell carcinoma: A single center experience of 18 cases. Clin. Genitourin. Cancer 2024; 22; 261–268. 10.1016/j.clgc.2023.11.008 38104031

[29] Trpkov K , Hes O , Bonert M *et al*. Eosinophilic, solid, and cystic renal cell carcinoma: Clinicopathologic study of 16 unique, sporadic neoplasms occurring in women. Am. J. Surg. Pathol. 2016; 40; 60–71. 10.1097/PAS.0000000000000508 26414221

[30] Kammerer‐Jacquet SF , Gandon C , Dugay F *et al*. Comprehensive study of nine novel cases of TFEB‐amplified renal cell carcinoma: An aggressive tumour with frequent PDL1 expression. Histopathology 2022; 81; 228–238. 10.1111/his.14683 35562857

[31] Whaley RD , Sill DR , Tekin B *et al*. Evaluation of 3,606 renal cell tumors for TFE3 rearrangements and TFEB alterations via fluorescence in situ hybridization, next generation sequencing, and GPNMB immunohistochemistry. Hum. Pathol. 2025; 159; 105797. 10.1016/j.humpath.2025.105797 40381702

[32] Argani P , Baraban E , Yaskiv O *et al*. Clinically sporadic folliculin ‐mutated renal epithelial neoplasms represent a mixture of True somatic folliculin ‐mutated and occult Birt‐hogg‐Dubé syndrome‐associated cases: Morphologic and molecular overlap with TSC/MTOR ‐mutated eosinophilic renal neoplasms and MiT family translocation renal cell carcinoma. Am. J. Surg. Pathol. 2025; 49; 859–872. 10.1097/PAS.0000000000002413 40321032

[33] Kwon JW , Tjota MY , Antic T . MTOR‐mutated eosinophilic renal cell carcinomas with loss of chromosome 1 and high metastatic potential: Further expanding the spectrum of TSC/mTOR pathway mutated renal tumors. Virchows Arch. 2025. 10.1007/s00428-025-04363-4. PMC13582412 41299159

[34] Zhang C , Duan Y , Xia M *et al*. TFEB mediates immune evasion and resistance to mTOR inhibition of renal cell carcinoma via induction of PD‐L1. Clin. Cancer Res. 2019; 25; 6827–6838. 10.1158/1078-0432.CCR-19-0733 31383732

[35] Walter B , Gil S , Naizhen X *et al*. Determination of the expression of PD‐L1 in the morphologic Spectrum of renal cell carcinoma. J. Cancer 2020; 11; 3596–3603. 10.7150/jca.35738 PMC7150459 32284756

[36] Zhang M , Xian J , Tang J *et al*. Clinicopathologic and molecular study of TFEB ‐altered renal cell carcinomas: Tumors with frequent PDL1 expression. Am. J. Surg. Pathol. 2026; 50; 84–102. 10.1097/PAS.0000000000002458 40704447

[37] Giunchi F , Degiovanni A , Daddi N *et al*. Fading with time of PD‐L1 immunoreactivity in non‐small cells lung cancer tissues: A methodological study. Appl. Immunohistochem. Mol. Morphol. 2018; 26; 489–494. 10.1097/PAI.0000000000000458 27801735

[38] Fernandez AI , Gaule P , Rimm DL . Tissue age affects antigenicity and scoring for the 22C3 immunohistochemistry companion diagnostic test. Mod. Pathol. 2023; 36; 100159. 10.1016/j.modpat.2023.100159 PMC10502188 36925070

[39] Smith SC , Yu J , Paul AK . A TFEB‐amplified renal cell carcinoma with Long‐term, complete immunotherapy response: Retrospective support for the value of molecular classification. Int. J. Surg. Pathol. 2024; 32; 539–542. 10.1177/10668969231185077 37415407

[40] Argani P , Mehra R . Renal cell carcinoma associated with tuberous sclerosis complex (TSC)/mammalian target of rapamycin (MTOR) genetic alterations. Mod. Pathol. 2022; 35; 296–297. 10.1038/s41379-021-00971-y 35046523

[41] Alesi N , Asrani K , Lotan TL , Henske EP . The Spectrum of renal “TFEopathies”: Flipping the mTOR switch in renal tumorigenesis. Physiology (Bethesda) 2024; 39; 359. 10.1152/physiol.00026.2024 PMC12312796 39012319

[42] Albertson D , Barry M , Liu T , Mahlow J , Sirohi D . Genotype phenotype correlation of renal tumors in the cancer genome atlas database. Int. J. Surg. Pathol. 2025; 33; 289–301. 10.1177/10668969241260236 39034146

[43] Nord AS , Lee M , King MC , Walsh T . Accurate and exact CNV identification from targeted high‐throughput sequence data. BMC Genomics 2011; 12; 184. 10.1186/1471-2164-12-184 PMC3088570 21486468

[44] Hayes JL , Tzika A , Thygesen H *et al*. Diagnosis of copy number variation by Illumina next generation sequencing is comparable in performance to oligonucleotide array comparative genomic hybridisation. Genomics 2013; 102; 174–181. 10.1016/j.ygeno.2013.04.006 23598253

[45] Du Z , Hu J , Chen G *et al*. Targeted next‐generation sequencing for copy number variation detection: Recent progress in clinical oncology. Chin. Med. J. 2026; 139; 1107–1109. 10.1097/CM9.0000000000004001 PMC13090049 41796055

